# Obese motorcycle riders have a different injury pattern and longer hospital length of stay than the normal-weight patients

**DOI:** 10.1186/s13049-016-0241-4

**Published:** 2016-04-14

**Authors:** Hang-Tsung Liu, Cheng-Shyuan Rau, Shao-Chun Wu, Yi-Chun Chen, Shiun-Yuan Hsu, Hsiao-Yun Hsieh, Ching-Hua Hsieh

**Affiliations:** Department of Trauma Surgery, Kaohsiung Chang Gung Memorial Hospital and Chang Gung University College of Medicine, No.123, Ta-Pei Road, Niao-Sung District, Kaohsiung City, 833 Taiwan; Department of Neurosurgery, Kaohsiung Chang Gung Memorial Hospital and Chang Gung University College of Medicine, Kaohsiung City, Taiwan; Department of Anesthesiology, Kaohsiung Chang Gung Memorial Hospital and Chang Gung University College of Medicine, Kaohsiung City, Taiwan

**Keywords:** Motorcycle-related injury, Obesity, Injury Severity Score, Mortality, In-hospital length of stay

## Abstract

**Background:**

The adverse effects of obesity on the physical health have been extensively studied in the general population, but not in motorcycle riders (includes both drivers and pillions). The aim of this study was to compare injury patterns, injury severities, mortality rates, and in-hospital or intensive care unit (ICU) length of stay (LOS) between obese and normal-weight patients who were hospitalized for the treatment of trauma following motorcycle accidents in a level I trauma center.

**Methods:**

Detailed data of 466 obese adult patients with a body mass index (BMI) ≥30 kg/m^2^ and 2701 normal-weight patients (25 > BMI ≥18.5 kg/m^2^) who had sustained motorcycle accident-related injuries were retrieved from the Trauma Registry System between January 1, 2009 and December 31, 2013. We used the Pearson’s chi-squared test, Fisher’s exact test, and independent Student’s *t*-test to analyze differences between the two groups.

**Results:**

Compared to normal-weight motorcycle riders, more obese riders were men and drivers as opposed to pillions. In addition, fewer obese motorcycle riders showed alcohol intoxication. Analyses of the patients’ Abbreviated Injury Scale (AIS) scores revealed that obese motorcycle riders presented with a higher rate of injury to the thorax, but a lower rate of injury to the face than normal-weight patients. In addition, obese motorcycle riders had a 2.7-fold greater incidence of humeral, 1.9-fold greater incidence of pelvic, and 1.5-fold greater incidence of rib fractures. In contrast, normal-weight motorcycle riders sustained a significantly higher rate of maxillary and clavicle fractures. Obese motorcycle riders had a significant longer in-hospital LOS than normal-weight motorcycle riders did (10.6 days vs. 9.5 days, respectively; *p* = 0.044), with an increase in in-hospital LOS of 0.82 days associated with every 10-unit increase in BMI. No statistically significant differences in Injury Severity Score (ISS), New Injury Severity Score (NISS), Trauma-Injury Severity Score (TRISS), mortality, percentage of patients admitted to the ICU, or LOS in the ICU were found between obese and normal-weight patients.

**Discussion:**

No differences of injury severity, mortality, and LOS in the ICU between obese and normal-weight motorcycle riders in this study may be partly attributed to the motorcycle injuries occur at relatively low velocity, considering that the riding of majority of motorcycles are forbidden on highways in Taiwan and that most traffic accidents occur in relatively crowded streets.

**Conclusion:**

Obese motorcycle riders had different injury characteristics and bodily injury patterns than normal-weight motorcycle riders. Moreover, they had a longer in-hospital LOS; this was particularly true for those with pelvic fractures. However, injury severity and mortality were not significantly different between the two groups.

## Background

Obesity is a chronic metabolic disorder that has become an epidemic and major health problem worldwide [1, 2]. The adverse effects of obesity on the physical health have been extensively studied in the general population. While it is known that obesity increases the risk for a variety of medical conditions including hypertension, diabetes mellitus, cardiac disease, and pulmonary thromboembolism [3], the effect of obesity on the injury pattern and outcome of the trauma patients remains unclear. Recent studies have indicated that obese trauma patients are more likely to require mechanical ventilation, develop multiple organ failure, and spend more time in the intensive care unit (ICU) [4]. In addition, prior studies of trauma patients have described an association between obesity and mortality [5, 6]. Whereas two studies showed that obese men had the highest mortality risk among the trauma patients [7, 8], others found no significant difference in mortality between obese and non-obese patients [9, 10], even after emergency surgery [11, 12].

Using a motorcycle as a means of transportation is becoming popular in many cities, as it is cheaper, easier, and more fuel-efficient. Motorcyclists are extremely vulnerable road participants and can suffer from severe and often fatal injuries. The increased use of motorcycles for recreation and the availability of more powerful motorcycles has led to an increased incidence of motorcycle fatalities and injuries [13]. In Taiwan, motorcyclists comprise a major portion of the trauma population [14]. Given the increase in body mass index (BMI) in the general population, it is not surprising that motorcycle riders’ weights have followed a similar trend [15]. Since the energy involved in an impact is directly proportional to both mass and velocity (squared), large unrestrained individuals are at higher risk of injury. An elevated BMI has been suggested to intensify the energy dissipated in a crash and therefore possibly increase the individual’s vulnerability to serious injury or death [15]. Mock et al. showed an odds ratio (OR) for death of 1.013 (95 % CI 1.007–1.018) for each kilogram increase in body weight [7]. In their study, the OR for sustaining an injury with an Injury Severity Score (ISS) ≥9 was 1.008 (95 % CI 1.004–1.011) for each kilogram increase in body weight [7].

The identification of high-risk injury patterns might lead to improved care in trauma patients who are admitted to the hospital [16, 17]. Gaining a better understanding of the epidemiology of motorcycle-related trauma is vital to integrate the knowledge of trauma care into the trauma system that has to cope with a rising number of obese patients. Therefore, this study investigated the injury characteristics, injury patterns, injury severities, and mortality rates of obese patients who were treated for injuries that they sustained in motorcycle accidents in a level I trauma center in southern Taiwan.

## Methods

This study was conducted at the Kaohsiung Chang Gung Memorial Hospital, a 2400-bed facility and level I regional trauma center that provides care to trauma patients primarily from South Taiwan. Approval for this study was obtained from the hospital’s institutional review board (IRB) before its initiation with the approval number 103-5015B. Given its observational nature, the requirements for written informed consent from each patient was waived by IRB. This retrospective study was designed to review all data added to the Trauma Registry System from January 1, 2009 to December 31, 2013 and select cases that met the following inclusion criteria: (1) adult patients aged 20–65 years, (2) obese patients with a BMI ≥30 kg/m^2^ and normal-weight patients with a BMI <25 but ≥18.5 kg/m^2^ according to the definition of the World Health Organization [18, 19], and (3) hospitalization for the treatment of trauma following a motorcycle accident. The data of patients who had sustained injuries in a motorcycle accident, including road and off-road motorcyclist accidents, were collected for further analysis.

We reviewed the data of all 16,548 registered hospitalized patients. Among these, 4773 patients (28.8 %) were adult motorcycle drivers and pillions (hereafter referred to as motorcycle riders) with validated BMI data. Detailed patient information was retrieved from the Trauma Registry System of our institution and included data on age, sex, vital signs at admission, injury mechanism, helmet use, Glasgow Coma Scale (GCS) upon arrival at the emergency department, Abbreviated Injury Scale (AIS) severity score for each body region, ISS, New Injury Severity Score (NISS), Trauma-Injury Severity Score (TRISS), in-hospital length of stay (LOS), LOS in the ICU, in-hospital mortality, and rates of associated complications. In addition, the pre-existed comorbidities and chronic diseases including diabetes mellitus (DM), hypertension (HTN), coronary artery diseases (CAD), and end-stage renal disease (ESRD) were identified. A blood alcohol concentration (BAC) of 50 mg/dL at the time of arrival at the hospital was defined as the cut-off value, as it represents the legal limit for drivers in Taiwan.

Data collected regarding the obese and normal-weight population of motorcycle riders were compared using the SPSS v.20 statistical software (IBM, Armonk, NY, USA). Pearson’s chi-squared tests, Fisher’s exact tests, and independent Student’s t-tests were used to analyze data as applicable. The ORs of the injuries and associated conditions sustained by obese and normal-weight patients were calculated with 95 % CIs. The adjusted odds ratios (AORs) and 95 % CIs for mortality were estimated through stepwise model selection of a multiple regression model that was adjusted by controlling the cofounding variables. Linear regression was used to evaluate the effect of the BMI on the in-hospital LOS. In the regression analysis, in-hospital LOS was used as the dependent variable and the BMI as the independent variable. All results are presented as means ± standard errors. A p-value <0.05 was considered statistically significant.

## Results

### Injury characteristics

**Fig. 1:**
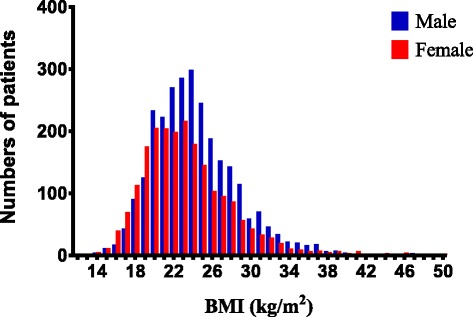
Body mass indices of the motorcycle riders who were admitted for the treatment of trauma by sex

Among the 4773 motorcycle riders, 466 (9.8 %) were obese, 1317 (27.6 %) overweight (30 > BMI ≥25 kg/m^2^), 2701 (56.6 %) normal-weight, and 289 (6.0 %) underweight (Fig. 1). Only obese patients (*n* = 466) and normal-weight patients (*n* = 2701) were chosen for further analysis. The mean ages of the obese and normal-weight patients were 40.7 ± 14.2 and 40.7 ± 14.2 years, respectively (Table 1). Of the 466 obese patients, 296 (63.5 %) were men and 170 (36.5 %) women. Of the 2701 normal-weight patients, 1476 (54.6 %) were men and 1225 (45.4 %) women. We found a significant statistically difference in the percentage of men vs. women between the groups (Fig. 1). In addition, there were significant higher incidence rates of the pre-existed comorbidities and chronic diseases including DM and HTN, but not CAD nor ESRD in the obese motorcycle riders.

**Table 1:** Demographics of motorcycle riders hospitalized for trauma

Variables	Obese	Normal	Odds ratio (95 %)	*P*
	BMI ≥ 30	25 > BMI ≥ 18.5		
	*n* = 466	*n* = 2701		
Gender				
Male	296(63.5)	1476(54.6)	1.4(1.18–1.77)	<0.001
Female	170(36.5)	1225(45.4)	0.7(0.57–0.85)	<0.001
Age	40.7 ± 14.2	40.7 ± 14.2	—	0.986
Mechanism, n(%)				
Motorcycle Driver	452(97.0)	2559(94.7)	1.8(1.03–3.13)	0.038
Motorcycle Pillion	14(3.0)	142(5.3)	0.6(0.32–0.98)	0.038
Helmet use, n(%)				
Yes	414(88.8)	2360(87.4)	1.2(0.84–1.57)	0.375
NO	42(9.0)	277(10.3)	0.9(0.62–1.22)	0.410
Unknown	10(2.1)	64(2.4)	0.9(0.46–1.77)	0.768
Alcohol > 50, n(%)	42(9.0)	336(12.4)	0.7(0.50–0.98)	0.035
GCS	14.3 ± 2.3	14.1 ± 2.5	—	0.197
≤8	21(4.5)	166(6.1)	0.7(0.45–1.15)	0.166
9–12	23(4.9)	127(4.7)	1.1(0.67–1.66)	0.826
≥13	422(90.6)	2408(89.2)	1.2(0.84–1.63)	0.363
AIS, n(%)				
Head/Neck	136(29.2)	887(32.8)	0.8(0.68–1.05)	0.119
Face	99(21.2)	767(28.4)	0.7(0.54–0.86)	0.001
Thorax	101(21.7)	389(14.4)	1.6(1.29–2.10)	<0.001
Abdomen	33(7.1)	195(7.2)	1.0(0.67–1.44)	0.915
Extremity	359(77.0)	1969(72.9)	1.2(0.99–1.57)	0.061
ISS	9.7 ± 8.1	9.3 ± 7.5	—	0.339
<16	376(80.7)	2231(82.6)	0.9(0.69–1.13)	0.318
16–24	58(12.4)	334(12.4)	1.0(0.75–1.36)	0.961
≥25	32(6.9)	136(5.0)	1.4(0.93–2.07)	0.103
NISS	11.5 ± 9.5	11.0 ± 9.2	—	0.258
TRISS	0.965 ± 0.100	0.964 ± 0.111	—	0.779
Mortality, n(%)	7(1.5)	32(1.2)	1.3(0.56–2.90)	0.566
LOS (days)	10.6 ± 10.9	9.5 ± 10.2	—	0.044
ICU				
Patients, n(%)	93(20.0)	492(18.2)	1.1(0.87–1.43)	0.371
<16	29(6.2)	162(6.0)	1.0(0.69–1.56)	0.850
16–24	37(7.9)	210(7.8)	1.0(0.71–1.47)	0.902
≥25	27(5.8)	120(4.4)	1.3(0.86–2.03)	0.200
LOS in ICU (days)	7.9 ± 7.3	7.0 ± 8.2	—	0.311
<16	6.4 ± 5.5	5.1 ± 4.6	—	0.175
16–24	6.4 ± 5.3	6.1 ± 5.0	—	0.745
≥25	11.7 ± 9.9	11.2 ± 13.4	—	0.853

Among the injured patients, 3011 (452 obese [97.0 %] and 2559 normal-weight [94.7 %] patients) were motorcycle drivers, whereas only 156 (14 obese [3.0 %] and 142 normal-weight [5.3 %] patients) were pillions (Table 1). Significantly more obese patients were motorcycle drivers and significantly less were pillions when compared to normal-weight patients (*p* = 0.038 for each group). Almost 98 % of patients wore helmets in both groups and the difference between these two groups was not statistically significant. A positive BAC was less frequent among obese than normal-weight patients (9.0 % vs. 12.4 %, respectively; *p* = 0.035).

### Injury severity

No significant differences in GCS scores (14.3 ± 2.3 vs. 14.1 ± 2.5, respectively; *p* = 0.197) and the distribution of proportion of patients at different levels of consciousness (GCS ≤8, 9–12, or ≥13) were found between obese and normal-weight patients (Table 1). Analysis of AIS scores revealed that obese patients had sustained significantly higher rates of thoracic injuries than normal-weight patients had (21.7 % vs. 14.4 %, respectively; p <0.001), while normal-weight patients had sustained significantly higher rates of facial injuries (28.4 % vs. 21.2 %, respectively; *p* = 0.001). No significant differences were found between obese and normal-weight motorcycle riders for ISS regardless of subtype of injury severity (9.7 ± 8.1 vs. 9.3 ± 7.5, respectively; *p* = 0.339), NISS (11.5 ± 9.5 vs. 11.0 ± 9.2, respectively; *p* = 0.258), TRISS (0.965 ± 0.100 vs. 0.964 ± 0.111, respectively; *p* = 0.779); and in-hospital mortality (1.5 % vs. 1.2 %, respectively; *p* = 0.566). After adjusting for the existed cofounding variables between these two groups of patients, which included comorbidity (DM and HTN), gender, positive BAC, AIS injures to the face or thorax, we found no significant differences were found between obese and normal-weight motorcycle (AOR: 1.14, 95 % CI: 0.49–2.67; *p* = 0.768). We found that obese patients had a significantly longer in-hospital LOS than normal-weight patients did (10.6 days vs. 9.5 days, respectively; *p* = 0.044).

The linear regression analysis that was used to evaluate the effect of BMI on in-hospital LOS revealed that BMI was positively associated in- hospital LOS, such that for every 10 unit increase in BMI an increase in in-hospital LOS of 0.82 days was detected (*p* = 0.033). No significant differences between obese and normal-weight motorcycle riders were found for the percentage of patients admitted to the ICU (20.0 % vs. 18.2 %, respectively, *p* = 0.371) or the LOS in the ICU (7.9 days vs. 7.0 days, respectively; *p* = 0.311), regardless of injury severity.

### Physiological response & procedures performed at emergency department

Upon arrival at the emergency department, obese patients exhibited higher ORs for presenting with worse heart (OR 1.3, 95 % CI 1.04–1.68; *p* = 0.021) and respiratory rates (OR 3.7, 95 % CI 1.19–11.21; *p* = 0.031) than normal-weight patients (Table 2). No significant differences were detected for other measures such as a GCS <13 or a systolic blood pressure (SBP) <90 mmHg were found between obese and normal-weight patients. In addition, no significant differences were found in the odds for requiring procedures at the emergency department (cardiopulmonary resuscitation, intubation, chest tube insertion, and blood transfusion) (Table 2).

**Table 2:** Worse physiological response on arrival upon and procedures performed at the emergency department

Variables	Obese	Normal	Odds ratio (95 %)	*P*
	BMI ≥ 30	25 > BMI ≥ 18.5		
	*n* = 466	*n* = 2701		
Worse physiology at ER, n(%)				
GCS < 13	44(9.4)	293(10.8)	0.9(0.61–1.20)	0.363
SBP < 90 mmHg	16(3.4)	69(2.6)	1.4(0.78–2.36)	0.278
Heart rate > 100 beats/min	105(22.5)	487(18.0)	1.3(1.04–1.68)	0.021
Respiratory rate < 10 or >29	5(1.1)	8(0.3)	3.7(1.19–11.21)	0.031
Procedures at ER, n(%)				
Cardiopulmonary resuscitation	1(0.2)	3(0.1)	1.9(0.20–18.63)	0.471
Intubation	6(1.3)	65(2.4)	0.5(0.23–1.23)	0.132
Chest tube insertion	8(1.7)	44(1.6)	1.1(0.49–2.26)	0.891
Blood transfusion	19(4.1)	70(2.6)	1.6(0.95–2.68)	0.073

### Injury patterns

Table 3 shows the injuries that were associated with the motorcycle accidents. A significantly higher odds of obese motorcycle riders vs. normal-weight patients sustained rib fractures (OR 1.5, 95 % CI 1.14–1.99; *p* = 0.004), humeral (OR 2.7, 95 % CI 1.91–3.83; p <0.001), and pelvic fractures (OR 1.9, 95 % CI 1.23–2.94; *p* = 0.004) than normal-weight patients. In contrast, a significantly lower odds of obese motorcycle riders sustained maxillary (OR 0.5, 95 % CI 0.33–0.72; p <0.001) and clavicle fractures (OR 0.6, 95 % CI 0.46–0.85; *p* = 0.003). The in-hospital LOS was significantly longer in obese patients with pelvic fractures than in normal-weight patients with pelvic fractures (24.6 days vs. 17.2 days, respectively; *p* = 0.019). For the above-mentioned five sustained injuries. No significant differences of in hospital LOS were found in the patients sustaining maxillary fracture, rib, clavicle, or humeral fractures (Table 4).

**Table 3:** Associated injuries of the motorcycle riders

Variables	Obese	Normal	Odds ratio (95 %)	*P*
	BMI ≥ 30	25 > BMI ≥ 18.5		
	*n* = 466	*n* = 2701		
Head trauma, n(%)				
Neurologic deficit	3(0.6)	28(1.0)	0.6(0.19–2.04)	0.611
Cranial fracture	31(6.7)	236(8.7)	0.7(0.51–1.10)	0.135
Epidural hematoma (EDH)	20(4.3)	152(5.6)	0.8(0.47–1.21)	0.240
Subdural hematoma (SDH)	47(10.1)	281(10.4)	1.0(0.70–1.34)	0.835
Subarachnoid hemorrhage (SAH)	45(9.7)	326(12.1)	0.8(0.56–1.08)	0.135
Intracerebral hematoma (ICH)	10(2.1)	73(2.7)	0.8(0.41–1.54)	0.487
Cerebral contusion	27(5.8)	174(6.4)	0.9(0.59–1.36)	0.596
Cervical vertebral fracture	6(1.3)	21(0.8)	1.7(0.67–4.15)	0.272
Maxillofacial trauma, n(%)				
Orbital fracture	10(2.1)	97(3.6)	0.6(0.31–1.14)	0.111
Nasal fracture	7(1.5)	51(1.9)	0.8(0.36–1.76)	0.566
Maxillary fracture	30(6.4)	334(12.4)	0.5(0.33–0.72)	<0.001
Mandibular fracture	12(2.6)	120(4.4)	0.6(0.31–1.04)	0.062
Thoracic trauma, n(%)				
Rib fracture	71(15.2)	288(10.7)	1.5(1.14–1.99)	0.004
Sternal fracture	0(0.0)	3(0.1)	—	1.000
Hemothorax	14(3.0)	51(1.9)	1.6(0.88–2.93)	0.117
Pneumothorax	7(1.5)	55(2.0)	0.7(0.33–1.62)	0.442
Hemopneumothorax	12(2.6)	40(1.5)	1.8(0.92–3.38)	0.086
Lung contusion	9(1.9)	36(1.3)	1.5(0.70–3.05)	0.313
Thoracic vertebral fracture	5(1.1)	18(0.7)	1.6(0.60–4.38)	0.369
Abdominal trauma, n(%)				
Intra-abdominal injury	9(1.9)	50(1.9)	1.0(0.51–2.14)	0.906
Hepatic injury	11(2.4)	74(2.7)	0.9(0.45–1.63)	0.640
Splenic injury	10(2.1)	41(1.5)	1.4(0.71–2.86)	0.320
Retroperitoneal injury	0(0.0)	5(0.2)	—	1.000
Renal injury	5(1.1)	17(0.6)	1.7(0.63–4.66)	0.357
Urinary bladder injury	0(0.0)	0(0.0)	—	—
Lumbar vertebral fracture	2(0.4)	29(1.1)	0.4(0.09–1.67)	0.304
Sacral vertebral fracture	5(1.1)	12(0.4)	2.4(0.85–6.93)	0.092
Extremity trauma, n(%)				
Scapular fracture	11(2.4)	74(2.7)	0.9(0.45–1.63)	0.640
Clavicle fracture	51(10.9)	443(16.4)	0.6(0.46–0.85)	0.003
Humeral fracture	50(10.7)	115(4.3)	2.7(1.91–3.83)	<0.001
Radial fracture	62(13.3)	291(10.8)	1.3(0.95–1.71)	0.109
Ulnar fracture	28(6.0)	148(5.5)	1.1(0.73–1.67)	0.645
Metacarpal fracture	25(5.4)	104(3.9)	1.4(0.90–2.22)	0.127
Pelvic fracture	28(6.0)	88(3.3)	1.9(1.23–2.94)	0.004
Femoral fracture	49(10.5)	240(8.9)	1.2(0.87–1.67)	0.259
Patella fracture	18(3.9)	79(2.9)	1.3(0.79–2.25)	0.278
Tibial fracture	49(10.5)	288(10.7)	1.0(0.72–1.36)	0.924
Fibular fracture	27(5.8)	136(5.0)	1.2(0.76–1.78)	0.494
Calcaneal fracture	32(6.9)	145(5.4)	1.3(0.88–1.93)	0.193
Metatarsal fracture	9(1.9)	65(2.4)	0.8(0.40–1.62)	0.531

**Table 4:** Length of stay in the hospital for the injuries (maxillary fracture, rib fracture, clavicle fracture, humeral fractures, and pelvic fracture) that showed different incidences in obese and normal-weight motorcycle riders

Variables	Obese	Normal	*P*
	BMI ≥ 30	25 > BMI ≥ 18.5	
	*n* = 466	*n* = 2701	
LOS (days)			
Maxillary fracture	10.2 ± 6.2	10.7 ± 8.1	0.713
Rib fracture	11.9 ± 11.9	10.8 ± 8.5	0.480
Clavicle fracture	8.9 ± 8.2	8.8 ± 10.1	0.971
Humeral fracture	10.4 ± 11.3	8.2 ± 7.4	0.212
Pelvic fracture	24.6 ± 15.8	17.2 ± 13.9	0.019

## Discussion

This study compared the patient demographics and injury characteristics of obese and normal-weight motorcycle riders who were hospitalized at a level I trauma center. More obese patients were men and motorcycle riders as opposed to pillions, and fewer presented with alcohol intoxication when compared to normal-weight patients. Obese motorcycle riders presented with different bodily injury patterns and had a longer in-hospital LOS when compared to normal-weight motorcycle riders.

It has been reported that obese trauma patients sustained more pelvic, rib, and lower extremity fractures, but fewer liver injuries, mandibular fractures, and cerebral injuries than those non-obese trauma patients [20]. Another study demonstrated similar injury patterns of fewer head, but more chest and lower extremity injuries [21]. Based on our analysis of the AIS, obese motorcycle riders presented with a higher rate of injuries to the thorax, but a lower rate of injuries to the face than normal-weight motorcycle riders. They also had a 2.7-fold greater incidence of humeral fractures, 1.9-fold greater incidence of pelvic fractures, and 1.5-fold greater incidence of rib fractures of obese motorcycle riders than normal-weight motorcycle riders did. In contrast, normal-weight motorcycle riders sustained a significantly higher odds of maxillary and clavicle fractures than obese motorcycle riders. However, no significant differences were found for ISS (regardless of injury severity subtype), NISS, TRISS, percentage of patients admitted to the ICU, or LOS in the ICU between obese and normal-weight patients.

Motorcyclist fatalities accounted for nearly 60 % of all driving fatalities in Taiwan between 2006 and 2008 [22]. Moreover, the authors of this study revealed an association between higher fatality rates and male sex, advanced age, unlicensed status, not wearing a helmet, riding after alcohol consumption, and alcohol consumption of more than 550 cc [22]. In the current study, the mortality rates in obese and normal-weight motorcycle riders were not significantly different (1.5 % vs 1.2 %, respectively). However, it is difficult to interpret the reported body of literature on the association between obesity and trauma, since reports vary widely in patient selection, stratification, and the definition of outcomes. In addition, the number of fatalities among obese motorcycle riders examined in this study was too small for statistical analysis. Considering that the riding of majority of motorcycles are forbidden on highways in Asian cities and that most traffic accidents occur in relatively crowded streets in these cities, we hypothesize that the motorcycle injuries that happen in the Southern Taiwan region occur at relatively low velocity [14]. This might partially explain why no differences were seen for injury severity, mortality, and LOS in the ICU between obese and normal-weight patients [4, 15].

Excessive alcohol use might contribute to excess body weight [23]. Unsurprisingly, a co-occurrence of obesity and alcohol use was found in approximately 34.7 % of men and 38.6 % of women in the United States [23]. In this study, a positive BAC was less frequent among obese than normal-weight patients (9.0 % vs. 12.4 %, respectively; *p* = 0.035). Further studies should investigate whether the lower frequency of alcohol intoxication in obese patients was caused by the drinking behavior of these patients or because higher alcohol levels are needed in individuals with a higher body fat mass to reach the same BAC as those with a normal fat mass [24]. Significant changes in pulse rate and SBP were found in motorcycle riders with weight loads of 0, 10, 15, and 20 kg [25]. The heavier the load, the greater was the change [25]. In this study, obese patients were more likely to have worse heart and respiratory rates in the emergency department than normal-weight patients. However, because of the lack of baseline data of these patients, it is hard to clarify the worse heart and respiratory rates of the obese patients are due to the existed baseline variances or a worse cardiovascular fitness during stress.

In our study, obese patients had a significantly longer in-hospital LOS than normal-weight patients did. However, this difference was not great, as an increase of in-hospital LOS of only 0.82 days was associated with every 10-unit increase in BMI. Of note, the in-hospital LOS was significantly longer in obese patients with pelvic fractures than in normal-weight patients with pelvic fractures (24.6 days vs. 17.2 days, respectively; *p* = 0.019). It has been reported that the mean duration of orthopedic surgery in morbidly obese patients was 30 % longer than in non-obese patients [4], and obesity was shown to be associated with more complications after the surgical treatment of pelvic ring injuries [26]. Moreover, medically stable obese patients were found to be almost twice as likely to experience delayed fracture fixation due to preference of the surgeon [4]. Specific challenges and complications after musculoskeletal injury, including difficulty in reducing acetabular fractures, have also been associated with obesity [27]. Therefore, our finding that obese motorcycle riders with pelvic fractures had a significantly longer in-hospital LOS than normal-weight patients with pelvic fractures did is not surprising.

The limitations of this study include the use of a retrospective design with its inherent selection bias and the lack of available data on the circumstances of the mechanisms of injury, the speed of the motorcycle during accidents, the effect of the type of motorcycle, the type of helmet material, or if any other protective gear such as knee braces were used. In addition, the statistical analysis may be underpowered regarding mortality due to a small number of fatalities among obese motorcycle riders. Last, lack of exposure data prevented the analysis of motorcycle-related injuries based on exposure-based risk (e.g., number of trips, hours of riding, and/or miles traveled).

## Conclusion

Compared to normal-weight adult motorcycle riders, obese riders presented with different injury characteristics and bodily injury patterns and had a longer in-hospital LOS; this was particularly true for those with pelvic fractures. However, injury severity, mortality, the percentage of patients admitted to the ICU, and the LOS in the ICU exhibited no statistically significant differences between obese and normal-weight motorcycle riders.
